# Recurrent cannabis-induced catatonia: a case report and comprehensive systematic literature review

**DOI:** 10.3389/fpsyt.2024.1332310

**Published:** 2024-01-18

**Authors:** Reza Moshfeghinia, Mehrnaz Hosseinzadeh, Sara Mostafavi, Roxana Jabbarinejad, Mahdi Malekpour, Elnaz Chohedri, Jamshid Ahmadi

**Affiliations:** ^1^Student Research Committee, Shiraz University of Medical Sciences, Shiraz, Iran; ^2^Research Center for Psychiatry and Behavior Sciences, Shiraz University of Medical Sciences, Shiraz, Iran; ^3^Substance Abuse Research Center, Shiraz University of Medical Sciences, Shiraz, Iran; ^4^Fasa Neuroscience Circle (FNC), Student Research Committee, Fasa University of Medical Sciences, Fasa, Iran; ^5^National Brain Center, Iran University of Medical Sciences, Tehran, Iran; ^6^The Ken and Ruth Davee Department of Neurology, Northwestern University Feinberg School of Medicine, Chicago, IL, United States; ^7^Institute for Multicultural Counseling & Education Services (IMCES), Los Angeles, CA, United States

**Keywords:** cannabis, catatonia, recurrence, addiction, electroconvulsive therapy

## Abstract

**Background:**

Catatonia presents itself as a complex neuropsychiatric syndrome, giving rise to various motor, speech, and behavioral challenges. It is noteworthy that approximately 10% of psychiatric hospital admissions can be attributed to this condition. It is imperative to note that cannabis-induced catatonia, while infrequent, has been linked to the use of marijuana. This connection has the potential to disrupt neurotransmitter systems, necessitating further research for a comprehensive understanding and effective treatment, particularly given the evolving trends in cannabis use. In this context, we shall delve into a unique case of recurrent cannabis-induced catatonia.

**Case presentation:**

A 23-year-old gentleman, who has previously struggled with substance use disorder, experienced the emergence of mutism, social isolation, and a fixed gaze subsequent to his use of cannabis. Remarkably, despite the absence of hallucinations, he exhibited recurrent episodes of catatonia. These episodes were effectively addressed through a combination of electroconvulsive therapy (ECT) and lorazepam administration. Notably, when the lorazepam dosage was gradually reduced to below 2 mg per day, the catatonic symptoms resurfaced; however, they promptly abated upon reinstating the medication. The diagnosis of cannabis-induced catatonia was established, and its management primarily involved a therapeutic approach encompassing ECT and lorazepam. It is pertinent to underscore that this catatonic condition can be directly linked to the individual’s cannabis usage.

**Conclusion:**

The connection between cannabis and catatonia is intricate and not entirely comprehended. Although cannabis possesses therapeutic advantages, it can paradoxically trigger catatonia in certain individuals. Multiple factors, such as genetics, cannabinoids, and neurotransmitter systems, contribute to this intricacy, underscoring the necessity for additional research.

## 1 Introduction

Catatonia is characterized by the existence of three or more of the following features: Catalepsy, waxy flexibility, stupor, agitation, mutism, negativism, posturing, mannerisms, stereotypies, grimacing, echolalia, and echopraxia (1). Approximately 10% of all admissions to acute psychiatric facilities stem from catatonia (2). The identification of psychiatric comorbidity in mood disorders, schizophrenia, substance-related disorders, and diverse medical conditions emphasizes the multifaceted etiological factors contributing to this phenomenon (3).

Cannabis-induced catatonia constitutes a rare yet potentially grave condition in which the utilization of cannabis, colloquially known as marijuana, becomes associated with the manifestation of catatonic symptoms (4). While, generally, cannabis is deemed to carry a lower risk of inducing catatonia when juxtaposed with other substances such as stimulants or hallucinogens (5), instances have been documented where the use of cannabis coincided with the emergence of catatonic symptoms. It is crucial to recognize that this occurrence remains relatively infrequent.

The neurobiological underpinnings of catatonia are intricate and not entirely elucidated, primarily due to its heterogeneous clinical manifestations and multifarious triggers. Dysregulation of neurotransmitter systems, encompassing dopamine, GABA, and glutamate, has been implicated in the pathophysiology of catatonia (6, 7). Tetrahydrocannabinol (THC), a constituent of cannabis, exerts an impact on the endocannabinoid system, potentially disrupting the regulatory functions of GABA and glutamate (8). Certain cases have established a link between cannabis use and catatonia, particularly in instances of heightened potency and frequency of use (4). Substantial research is warranted concerning cannabis-induced catatonia, with a specific focus on comprehending its risk factors and treatment modalities. It is noteworthy that the shifting landscape of cannabis use in the United States may exert an influence on its prevalence. Extended longitudinal studies are imperative due to our limited comprehension of its prognosis and long-term repercussions.

There exist scenarios in which cannabis-induced catatonia transpires as an isolated event, while in other instances, it recurs intermittently. Within this narrative, we elucidate an atypical case of recurrent cannabis-induced catatonia.

## 2 Case presentation

A solitary 23-year-old male, recognized for his history of polysubstance use disorder, sought assistance at an outpatient clinic. His visit was prompted by a constellation of concerns, including mutism, reduced oral intake, self-imposed social isolation, persistent insomnia, and a sustained fixed gaze that had persisted over the preceding 10 days. The patient was accompanied by his family, who brought him to the outpatient clinic. These symptoms had surfaced subsequent to his recent use of cannabis. It is noteworthy that, despite these distressing manifestations, no evidence of delusions, auditory or visual hallucinations was discernible.

Upon the patient’s initial encounter, and after the alleviation of symptoms we undertook a meticulous evaluation of the patient’s mood to ascertain any indications of depressed mood, manic states, or mood fluctuations, we noted the presence of a partially elated mood as the only remarkable feature. With regard to anxiety-related symptoms, no discernible evidence of anxiety, excessive worry, panic attacks, or phobias was detected. Importantly, his vital signs remained well within the normal range, and there were no apparent signs of physical injury or trauma.

A review of his medical history revealed three prior admissions stemming from catatonic episodes, with the patient having undergone eight sessions of electroconvulsive therapy (ECT) over the past 2 years. The specifics of these previous hospitalizations are detailed in Table 1. Interestingly, as reported by his family members, each of these episodes had commenced prior to his cannabis use and subsequently resolved following ECT treatment. It is of particular note that during the intervals between hospitalizations, the patient remained symptom-free despite abstaining from his medications.

**Table 1:** Case report timeline.

Date of admission	Days at the hospital	Summaries from history and laboratory findings	Diagnosis/treatment	Bush-Francis Catatonia Rating Scale
August 2021	26	Mutism, poor oral intake, depressed mood, anxiety, anhedonia, social isolation, agitation, aggression, negativism, self-care deficit Urine toxicology: positive for clonazepam	Substance withdrawal/sertraline, bupropion, aripiprazole, lithium, olanzapine, 2 ECT* sessions	NA
October 2022	21	Mutism, poor oral intake, immobility, insomnia, anxiety, paradoxical laughter, fixed gaze, aggression, irritability, waxy flexibility Urine toxicology: positive for methadone	Substance-induced psychotic disorder and cluster B personality/lorazepam, risperidone	NA
November 2022	20	Mutism, poor oral intake, immobility, hypersomnia, aggression, irritability, self-care deficit	Cannabis-induced psychotic disorder/lorazepam, olanzapine, 6 ECT* sessions	NA
June 2023	27	Mutism, poor oral intake, social isolation, insomnia, fixed gaze, elated mood Urine toxicology: positive for cannabinoids	Cannabis-induced catatonia/lorazepam, olanzapine, 8 ECT* sessions	8
August 2023	19	Mutism, poor oral intake, closed eye, negativism, incontinency Urine toxicology: positive for cannabinoids	Cannabis-induced catatonia/lorazepam, 6 ECT* sessions	7

^*^ECT, electroconvulsive therapy; NA, not applicable.

Given the patient’s current presentation marked by stupor, mutism, and negativism, we arrived at a diagnosis of retarded catatonia in accordance with the criteria set forth in DSM-5. The patient’s score on the 23-item Bush-Francis Catatonia Rating Scale was duly documented as 8. Consequently, he was admitted to the psychiatric unit for further evaluation and management.

Comprehensive laboratory investigations, including a complete blood count, electrolyte levels, liver function tests, urine analysis, and thyroid function tests, all returned within normal limits. The electrocardiogram yielded unremarkable results, and magnetic resonance imaging (MRI) revealed no structural abnormalities. Notably, in this catatonic presentation, the urine toxicology analysis confirmed the exposure to cannabinoids by returning positive for THC.

Treatment commenced with lorazepam at a dose of 1 mg, administered orally three times daily. However, after 72 h, with no discernible improvement in the patient’s condition, a decision was made to initiate ECT. Following two sessions of ECT, a marked improvement in the patient’s condition was observed, and his catatonic symptoms began to abate. Subsequently, the lorazepam dosage was reduced to 2 mg daily, administered in divided doses of 0.5 mg, 0.5 mg, and 1 mg.

In light of persistent disorganized behavior, the patient ultimately underwent a total of 8 ECT sessions over an 18-day period. Additionally, he was commenced on olanzapine at a dose of 5 mg orally each night at bedtime (QHS), later adjusted to 2.5 mg orally QHS. Despite two unsuccessful attempts to taper the lorazepam dosage, it was observed that the patient’s catatonic symptoms resurfaced whenever the lorazepam dosage fell below 2 mg daily, and promptly abated upon reinstating the 2 mg dosage.

Consequently, he was discharged on the 27th day, maintaining a daily lorazepam dose of 2 mg. However, following his discharge, the patient once again exhibited symptoms of mutism, diminished oral intake, negativism, closed eye, and incontinence following the use of cannabis. In accordance with the criteria delineated in DSM-5, a diagnosis of catatonia was reaffirmed, attributable to the presence of stupor, mutism, and negativism. The initial severity of his symptoms was rated as 7, based on the 23-item Bush-Francis Catatonia Rating Scale.

As a result, he was readmitted to our hospital, where a urine toxicology test once again confirmed the presence of cannabinoids. Following 3 sessions of ECT, his symptoms demonstrated notable improvement; nevertheless, 3 additional ECT sessions were deemed necessary. Subsequently, the patient was finally discharged after 19 days, devoid of any discernible symptoms. It was deduced that the periodic catatonic presentations experienced by the patient were inextricably linked to his cannabis use.

## 3 Discussion

In this study, we present a case of recurrent catatonia. Despite exhaustive medical and psychiatric assessments, we have been unable to elucidate the origin of this catatonic state within the framework of medical illnesses and the common psychiatric disorders often associated with catatonia, such as mood disorders and psychoses. The patient and his family have consistently denied any hallucinations, delusions, or significant mood disturbances experienced by the patient, with the exception of hypomania. Structural evaluations of the patient’s brain through both CT and MRI scans have revealed no discernible abnormalities. The sole substantial stressor evident in the patient’s history and clinical presentation is their chronic multi-drug addiction, notably, a noteworthy increase in natural cannabis consumption preceding the catatonic episodes. It is worth noting that the catatonia in this patient has shown resistance to oral and intravenous benzodiazepine treatment, necessitating the utilization of ECT to achieve significant symptom relief. Furthermore, it is noteworthy that the severity of catatonic episodes has exhibited a progressive worsening trend, with subsequent recurrences occurring at lower reported cannabis dosage levels. The causality assessment between cannabis use and the occurrence and recurrence of catatonia in this case, as evaluated using the World Health Organization - Uppsala Monitoring Centre (WHO-UMC) causality tool, is considered to be probable or likely (9).

### 3.1 Literature review

Although cannabis-induced catatonia is a severe and potentially life-threatening condition, considering its widespread use and its legalization for medical purposes in numerous clinical settings, the existing literature on this condition remains incomplete and inconsistent. A recent comprehensive systematic review, spanning 19 years up to 2020, revealed 14 published cases across 11 studies that establish a relationship between catatonia and cannabis or synthetic cannabinoids (SCs) (4). Furthermore, a cross-sectional study conducted by Yeoh et al. (5) examined electronic health records in London. This study successfully correlated 5.1% of catatonic episodes with substance-related causes, with the majority of cases (68 out of 108) attributed to cannabis use. These cases were observed either in the context of acute intoxication alongside chronic use (24 out of 68) or in the absence of chronic use (7 out of 68). Importantly, a significant portion of these cases showed no evidence of intoxication or withdrawal (37 out of 68) (5).

**Figure 1:**
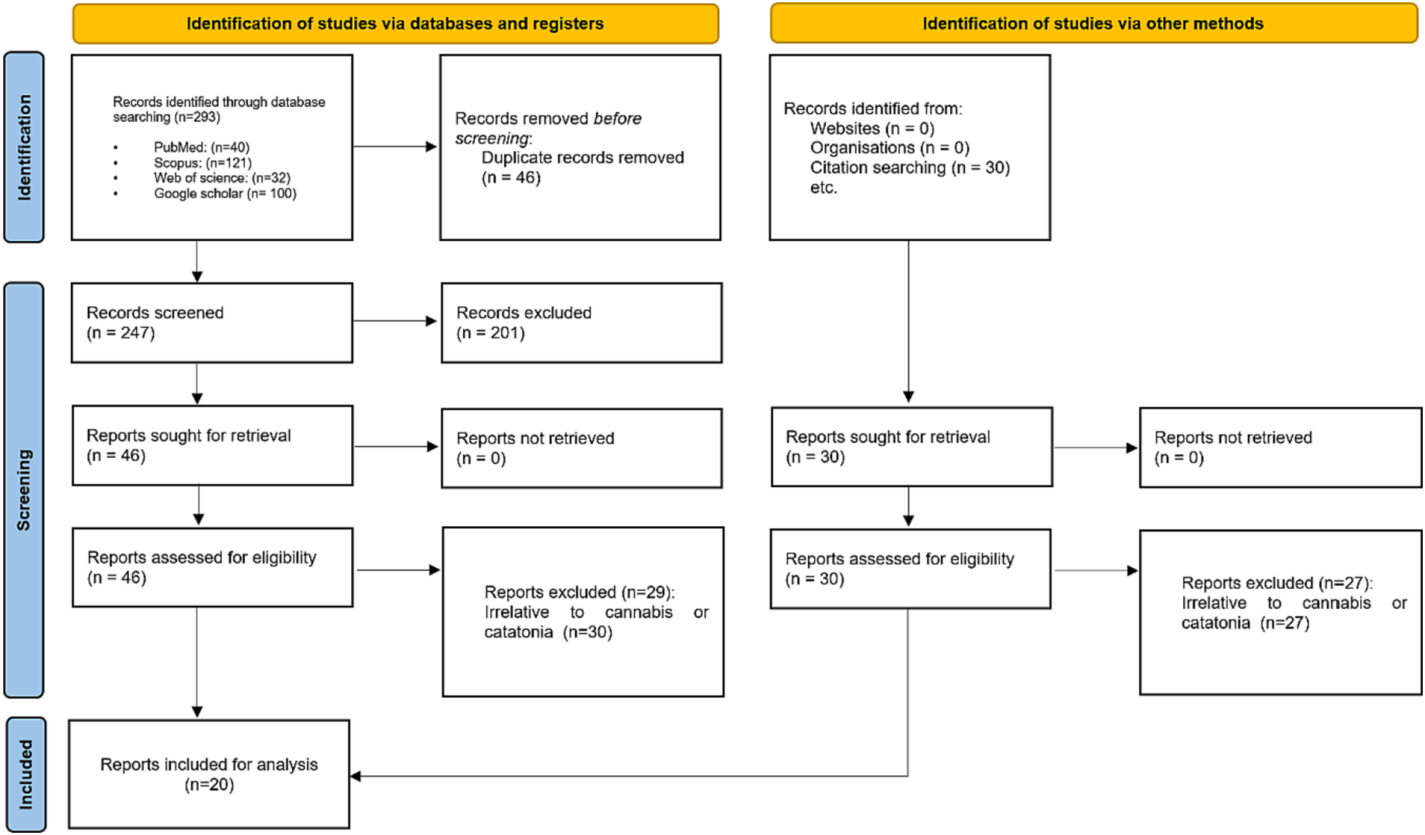
PRISMA flow diagram used for comprehensive literature search on cannabis-induced catatonia case reports.

A comprehensive literature review was conducted to investigate similar cases exploring the correlation between cannabis use and catatonia. English publications up to December 2023 were retrieved by querying four electronic databases (PubMed, Scopus, Web of Science, and Google Scholar). The search involved employing different combinations of keywords, such as “cannabis” AND “catatonia,” without imposing any restrictions on these terms or their synonyms. Detailed search methodologies for each database can be found in the Supplementary materials. To identify potentially relevant articles, we explored the citations of included studies. Our focus was on reviewing case studies (case reports or case series) specifically addressing cannabis-induced catatonia. The PRISMA flow diagram in Figure 1 illustrates the process. The initial search yielded 293 articles based on the specified keywords, with EndNote automatically eliminating 46 duplicates. After screening titles and abstracts, 247 articles were excluded, resulting in 46 articles for further consideration. Following a thorough examination of full texts, 29 articles were excluded, leaving a final selection of twenty studies for inclusion in our review.

Twenty-six patients out of twenty studies were identified, and their findings have been summarized in Table 2. Among these 26 cases, only 4 were female, constituting 84% of the sample who were male. The mean age of the patients was 22.24 ± 7.17 years old, ranging from 15 to 36 years old. Ten out of 25 cases involved pediatric patients. Additionally, it was reported that 11 cases had a history of using SCs.

**Table 2:** Overview of previous reported case with cannabis-induced catatonia.

Author	Country	Sex/Age	Intoxication	Psychiatric history	BFCRS score	Cannabis type	Recurrence of catatonia	Chronicity of use and catatonia	UTS	Symptoms	Treatment
Bajaj et al. (10)	India	Male/30	Yes	Chronic cannabis use	NR	NR	Yes; 5 episodes during last 7 years. All episodes proceeded by an increase in cannabis use	Subacute	NR	Mutism, negativism, posturing and poor oral intake	Lorazepam
Cohen et al. (11)	USA	Female/16	Yes	NR	NR	SC	1st presentation was reported.	Acute	+	AMS, mutism, vertical nystagmus, slightly rigid lower extremities, unresponsive to verbal and painful stimuli	Diphenhydramine, Lorazepam
	USA	Male/18	Yes	NR	NR	SC	1st presentation was reported.	Acute	−	Agitated, diaphoretic, headache, dizziness, tachycardia.	No benefit from lorazepam, but Diphenhydramine was effective in hours.
	USA	Male/16	Yes	Previously healthy	NR	SC	1st presentation was reported.	Acute	−	AMS, Hallucination, agitation, frozen face and dysarthric with pressured speech, dystonic, confused, conjunctival injection. Hypertonic and hyperreflexia extremities	Lorazepam.
Leibu et al. (12)	USA	Male/36	Yes	Schizophrenia, and chronic cannabis use	NR	SC	1st presentation was reported	Subacute	NR	Paranoia, auditory hallucination, disorganized thought with loosening of association, labile mood, pressured speech, bradykinesia and posturing, rigidity, waxy flexibility, and mutism.	Previous medical regimen (clozapine and perphenazine). lorazepam without effect, ECT eventuate rapid and dramatic response
Haro et al. (13)	Spain	Female/19	Yes/subacute or chronic	Chronic opioid and cannabis use	NR	SC	1st presentation was reported	Subacute	−	Visual hallucination, depersonalization and derealization, catatonia like symptoms: akinetic walking, rigid position similar to the praying mantis position	lorazepam, biperiden, and aripiprazole.
Smith et al. (14)	USA	Male/17	Yes	No history of psychosis		K2 (SC)	1st presentation was reported	Subacute			Lorazepam with limited benefits, ECT, Olanzapine
Caudron et al. (15)	France	Male/32	No/cannabis withdrawal	Mild ID (IQ = 67) chronic cannabis use, −ive for multidrug addiction	39/69	NR	1st presentation was reported	Subacute	−	Catatonia (mutism, confusion, eating avoidance, apathy, psychomotor agitation, catalepsy, waxy flexibility, plastic hypertonia, …)	Clonazepam, then lorazepam, Memantine
Håkansson et al. (16)	Sweden	Male/17	Yes	Chronic illicit drug use	NR	NR	At least 2 episodes of catatonic symptoms was reported	Subacute	+	Paranoid delusion, hallucination, agitation, mutism, depression, delusion of reference, withdrawal, bizarre posturing, and inappropriate laughs, faecal and urine incontinence	Diazepam, ECT, risperidone.
Khan et al. (17)	USA	Male/21	Yes	untreated childhood ADHD	NR	SC	1st presentation was reported	Chronic	+	Isolation, lack of attention to grooming and hygiene and unable to have oral intake independently for 1 month, mutism, self-talk, inappropriate laughter	Lorazepam, Risperidone switched to Aripiprazole
	USA	Male/17	Yes	previously healthy	NR	SC	1st presentation was reported	Subacute	−	Psychosis, partial mutism, echolalia, muscle rigidity and bradykinesia, euphoria, grandiosity, paranoia, disorganization and decreased sleep	Lorazepam improved speech and thought, Olanzapine with limited benefits, then Valproic acid with significant clinical benefits.
Roberto et al. (18)	USA	Male/18	Yes	Chronic SC use	NR	SC inhalation	Yes: 2 episodes, 2 weeks apart. Both happened after relapsed SC usage.	Subacute	+	Insomnia, elated mood, paranoia, delusion, and hallucination, agitation, disorganized behaviour progressive rigidity, mutism, and cognitive slowness	Lorazepam, risperidone benztropine
Pierre et al. (19)	USA	Male/17	Yes	Two years of cannabis use	NR	Concentrated cannabis extracts (wax)	1st presentation was reported	NR	−	Paranoid delusion, insomnia that started AMS, agitated, fever, tachycardia, hypertension, diaphoresis, and photophobia.	Risperidone
	USA	Male/26	Yes	Chronic cannabis use for social anxiety and obsessions	NR	Concentrated cannabis extracts (wax)	1st presentation was reported	NR	+	Paranoid delusion, restless, confused, bizarre behaviour.	Olanzapine changed to Risperidone Catatonia managed with lorazepam
Keller et al. (20)	USA	Male/34	Yes	Chronic substance use, TBI, PTSD	NR	Concentrated cannabis extracts (wax)	1st presentation was reported	Subacute	+	Labile mood, agitation, wild gesticulation, paranoia, staring, restricted affect, disorganization, depersonalization, derealization, incoherent speech, odd behaviour, psychomotor agitation, war related hallucinations.	Risperidone
Williams et al. (21)	Georgia	Male/19	Yes	Chronic use of Marijuana	NR	Spice (SC)	Relapse 9 days after discharged and other several times in next few months	Subacute	NR	Mutism, reduced oral intake, inattention to ADL, negativism, blunted affect, severe psychomotor retardation, bladder and bowel incontinency	Lorazepam with limited benefits, ECT with rapid response only after 1st session. Olanzapine
Bulbena-Cabre et al. (22)	USA	Male/50	Yes	Previously healthy	NR	K2, synthetic cannabinoid	1st presentation of catatonia was reported	NR	−	Rigidity, AMS, mutism, posturing	Midazolam, lorazepam, dexamethasone
Manning et al. (23)	USA	Male/20	Yes	Previous diagnoses of mania, cannabis use, GAD	19	Concentrated cannabis extract (vaping cannabis oil)	1st presentation of catatonia was reported, (2nd for psychosis and mania)	Subacute	+	Severe anxiety, poverty of thought, bizarre posturing, mutism, AMS withdrawal, staring, stereotypy, hallucination, delusion	Lorazepam lithium, sertraline.
Mekala et al. (24)	USA	Female/22	Yes	Chronic cannabis use	NR	Smoke	1st presentation was reported	Chronic	+	Depressed mood, suicidal ideation, psychosis, bizarre behaviour, mutism, thought block	Lorazepam, sertraline changed to duloxetine, and olanzapine
	USA	Female/33	Yes	Schizophrenia, and depression	NR	Resin	1st presentation was reported	Acute	NR	inability to do personal care, bizarre behaviour, worsening of auditory hallucination, mutism	Home medication (Olanzapine)
Sheikh et al. (25)	USA	Male/35	Yes	Chronic cannabis and opioid use, MDD, and suspected history of schizophrenia	12 and 18	NR	1st presentation was reported	Acute on chronic	+	AMS, hypertension, tachycardia, diaphoresis erratic behaviour, selective mutism, agitation, and periodic rigidity in all extremities, waxy flexibility (malignant catatonia)	Midazolam, antihypertensive and IV fluids. Then lorazepam and quetiapine,
Gouse et al. (26)	USA	Male/15	Yes	Non-suicidal self-injuries behavior	7	Vaping TKO most days of week for a year	Yes, twice. Both following increase in cannabinoid use	Chronic	+	Aggressive behaviour, auditory hallucination, paranoia, hyper religiosity, tachycardia, hypertension, mydriasis, psychomotor retardation, hyperreflexia, poor eye contact, monotonic speech, flat affect, mutism, rigidity, waxy flexibility, staring, immobility and posturing, catalepsy	Lorazepam and hydralazine.
	USA	Male/16	Yes	ADHD, and history of cannabis smoking	8	Vaping cannabis the day before admission	Yes, twice	Acute	+	AMS, hypertension, dizziness, hallucination writhing motion of head and neck, rigidity, staring, mutism, social withdrawal and autonomic instability	Lorazepam
Palkar et al. (27)	USA	Male/16	Yes	Chronic cannabis use	31/69	Vaping natural cannabis	1st presentation was reported	Subacute	+	Disorganized behaviour, staring, poor oral intake, mutism, rigid posturing, waxy flexibility, catalepsy, grimacing, hallucination, paranoia, hyperreligious	Lorazepam, Risperidone
Gauthier et al. (28)	USA	Male/15	Yes	Chronic cannabis use, mild depression, and remote history of seizures	3/69	Vaping SC	1st presentation was reported	NR	+	Progressive global weakness started from left leg, hypokinesia, AMS, minimal speech, staring	Lorazepam
Charlesworth et al. (29)	UK	Male/early 30s	Unreliable	Multi-drug addiction including cannabis use	26/69	Smoke	Recurrent, over 5 episodes of catatonia relapses- progressively milder	NR	−	Withdrawal, salivary dribbling, psychomotor retardation, urinary incontinence, mutism, AMS, staring, UTS was + only in the first admission. However, no temporal relationship to substance use or withdrawal was reported	Lorazepam, ECT

Chronicity of cannabis use and occurrence of catatonia: under 14 days defined as acute, 2 weeks-6 months as subacute and more than 6 months as chronic. BFCRS: Bush-Francis Catatonia Rating Scale; UTS: Urine toxicology screening; NR: not reported; AMS: Altered Mental Status; ECT: Electroconvulsive Therapy; ADL: activities of daily living; ID: Intellectual Disability; ADHD: Attention-Deficit/Hyperactivity Disorder; SC: Synthetic Cannabinoid; UDS: Urine Diagnostic Screening; EPS: Extrapyramidal Symptoms; GAD: Generalized Anxiety Disorder; MDD: Major Depressive disorder; TBI: Traumatic Brain Injury; PTSD: Post Traumatic Stress Disorder.

Recurrent catatonia, as observed in this case, is relatively uncommon and is not frequently reported in the literature. The majority of catatonia cases are typically episodic and resolve with appropriate treatment. The prevalence of recurrent catatonia remains uncertain due to its rarity, but it underscores the importance of studying such cases to uncover potential contributing factors. One study with a small sample size estimated catatonia recurrence to be 38.5% in bipolar disorder, based on subjective reports (30). Lin et al. performed a longitudinal study and, over 15 years, identified 30 cases of catatonia recurrences and relapses in their center, with the majority being related to a schizophrenia diagnosis (63.3%), and approximately 23.4% of patients had mood disorders. They examined the effect of a Benzodiazepine-based treatment protocol, and 79.4% of them had a full response, as in another study where almost all the patients with catatonia responded to the protocol, suggesting that recurrent catatonia may require more intervention or maintenance therapy after discharge (31, 32). Some studies claim that recurrent catatonia can be explained in the recurrent picture of mood disorders, and an underlying mood disorder may be masked behind catatonia. Due to the historical definition of catatonia, many may relate it to schizophrenia (33, 34). The recurrence of catatonia in the context of substance use, especially cannabis, is less demonstrated in the literature. Yeoh et al. (5), examined electronic health records of catatonia episodes and found an event rate of 1.46 in non-substance-related catatonia (94.9% of patients) and an event rate of 1.08 in substance-related catatonia (5.1% of patients) (5). Some of the reviewed case reports in this study provided reports of recurrence and relapses in the context of cannabis-induced catatonia (10, 16, 18, 21, 26, 29) (see Table 2). Recurrent episodes of catatonia may be a sign of an underlying psychiatric condition or triggered by various factors, including substance use or stressors. However, the pattern and pathophysiology of catatonia recurrence are under recognized in the literature. Charesworth et al. reported a similar case to our presented case, but in contrast to ours, the severity of catatonic episodes progressively became milder, and the treatment response was more desirable in the latest episodes (29).

### 3.2 Mechanisms for cannabis-induced catatonia

Cannabis has garnered growing interest for its potential therapeutic applications, particularly in the realm of movement disorders. A meta-analysis has concluded that cannabidiol (CBD) exerts a significant therapeutic effect on the dyskinesia and dystonia associated with Parkinsonism, and there is moderate evidence supporting its promising effects on tic disorders such as Tourette’s syndrome (35). However, another systematic review failed to provide a definitive conclusion regarding the impact of cannabis on dystonia or other motor symptoms (36). Notably, cannabis-induced catatonia presents a paradoxical situation. While cannabis is often associated with neuropsychiatric effects (37), the occurrence of cannabis-induced catatonia primarily involving motor symptoms rather than concurrent psychosis adds complexity to our comprehension. This paradox underscores the intricate interplay between cannabinoids, neural pathways, and neuropsychiatric responses, underscoring the need for further exploration and research into both the therapeutic potential and the enigmatic facets of cannabis usage in neurological and psychiatric disorders.

Research suggests that dysregulation of the endocannabinoid system may contribute to the development of catatonia in cannabis intoxication (28). The endocannabinoid system plays a critical role in motor control, as evidenced by the abundance of CB1 receptors in basal ganglia and cortical areas that modulate motor control (38). Delta-9-tetrahydrocannabinol (THC), the primary psychoactive component in cannabis, acts as a partial agonist of the CB1 receptor and can disrupt the normal activity of the endocannabinoid system (22, 38). Karniol et al. demonstrated that intraperitoneal injection of THC can induce catatonia in a dose-dependent pattern, and CBD can partially mitigate this effect (39).

Dysregulation of the endocannabinoid system is associated with a reduction in the GABAergic inhibition. The alteration in the GABAergic pathways, coupled with an increase in glutamatergic activity, can disrupt the normal functioning of the dopamine system, ultimately contributing to the manifestation of catatonia (7). The substantiation of GABAergic dysfunction’s involvement in the manifestation of catatonia is bolstered by limited observations illustrating substance-related catatonia, wherein baclofen, an agonist acting on the GABA system with effects on the central nervous system, can be associated to manifestation of catatonia (40). Catatonia has a complex pathogenesis and in the literature is associated with diverse set of mechanisms and comorbidities including systemic diseases such as autoimmune thyroiditis Ali et al. has found 13 cases in the literature with autoimmune thyroiditis and catatonia, they discussed that those systemic conditions may be underdiagnosed in the clinical settings (41).

The enduring consequences of cannabis utilization and the recurrence of cannabis-induced catatonia might possibly be correlated with the modulation of N-methyl-D-aspartate (NMDA) receptors. Anti-NMDA receptor encephalopathy is an autoimmune disease that most patients present with catatonia in the advanced stages of this disease, and has provided substantial insights into biological underpinning of catatonia (42).Cannabis, predominantly due to its psychoactive component THC, possesses the capacity to impact glutamate neurotransmission, a pivotal element in the functioning of NMDA receptors. Prolonged cannabis consumption has been connected to modifications in the density and sensitivity of NMDA receptors, potentially disrupting the delicate equilibrium of excitatory and inhibitory neurotransmission (37, 43).Chronic cannabis use is associated with instantiating neurodegenerative processes, a case report by Moshfeghinia et al. described a case with early-onset frontotemporal dementia following chronic cannabis use (44). These long lasting changes in brain structure and chemistry including perturbation in the glutamatergic system could instigate persistent alterations in neural circuitry and plasticity, thereby contributing to the continuation of catatonic symptoms beyond the acute intoxication phase (37, 42).

**Figure 2:**
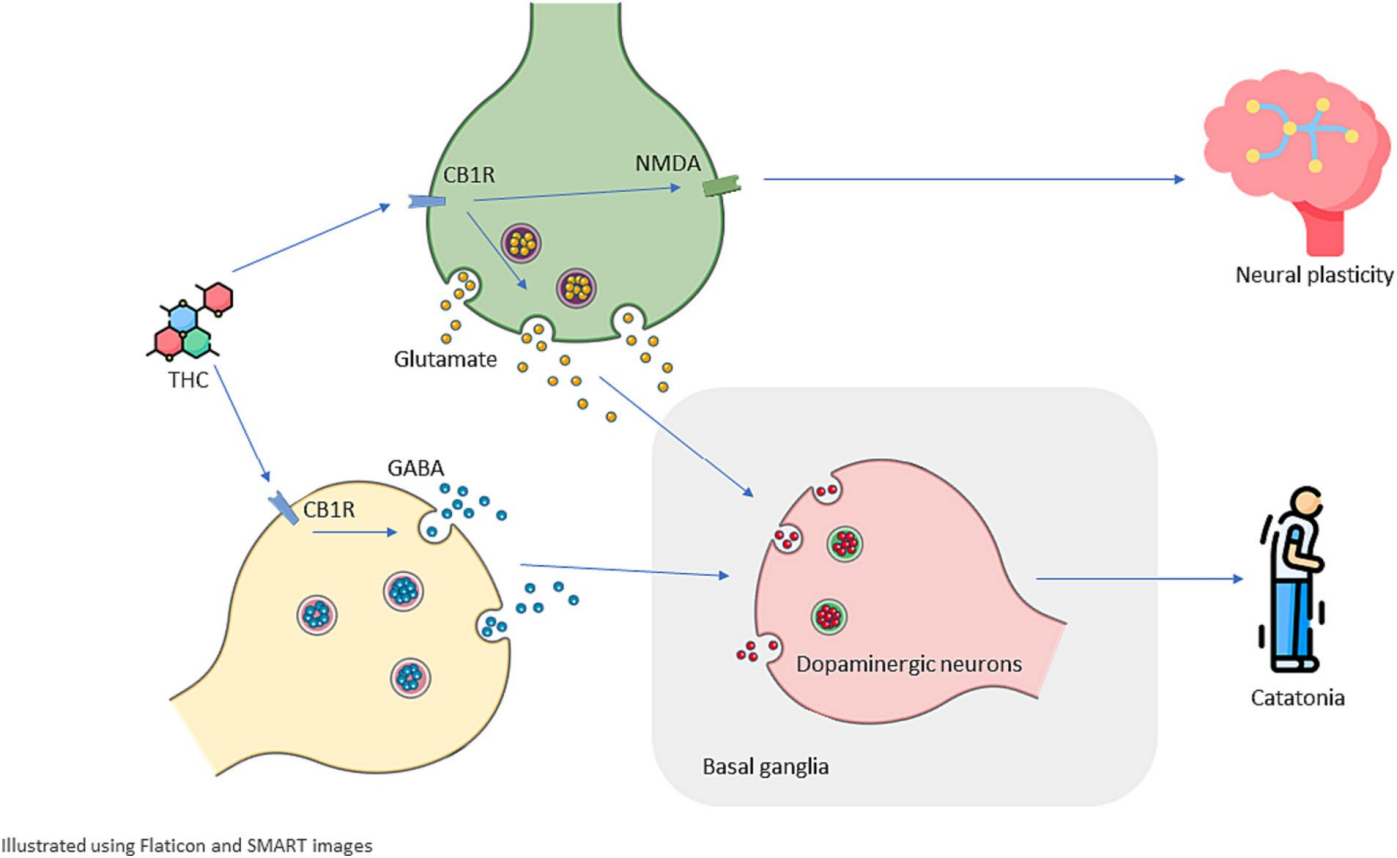
Schematic illustration of possible mechanisms underlying cannabis-induced catatonia and its recurrence. Delta-9 tetrahydrocannabinol (THC) acts as a partial agonist for CB1R (type 1 cannabinoid receptor) that can be found in the endocannabinoid system including both Glutamatergic and GABAergic neurons. THC disrupts excitatory-inhibitory balance and dysregulated Glutamatergic and GABAergic activity dysregulate Dopaminergic activity in basal ganglia which plays a significant role in motor control, this dysregulation can manifest as catatonia. Cannabinoids are associated with changes in the density and sensitivity of NMDA receptors which can depict how THC and cannabinoids can cause long-lasting effects on brain structure and chemistry and increase a patient’s susceptibility to present with recurrent catatonia.

While cannabis use is indeed prevalent, the occurrence of cannabis-induced catatonia remains rare. This rarity can be attributed to the intricate interplay of multiple factors. Firstly, individual susceptibility plays a pivotal role, with genetic predispositions and the diathesis-stress model contributing to varying responses among users (20, 38). It’s important to note that not everyone who uses cannabis will develop catatonic symptoms. Furthermore, the psychoactive compound THC, which is abundant in many cannabis strains, is often associated with neuropsychiatric effects. However, the presence of CBD, another prominent cannabinoid in cannabis, may serve as a protective factor. CBD is believed to counterbalance some of the adverse effects of THC, potentially mitigating the risk of developing catatonia in susceptible individuals (45). This intricate interaction of genetic, environmental, and pharmacological variables underscores the complexity of cannabis-induced catatonia, rendering it an infrequent manifestation despite the widespread use of cannabis. Figure 2 summarize the mentioned pathways that cannabis can potentially change to induce catatonia.

### 3.3 Limitations

This case report encountered numerous challenges and limitations. For instance, non-convulsive status epilepticus, a potential differential diagnosis for sudden onset catatonia (46), remained unexplored, with no EEG study documented during the patient’s admissions. Additionally, our case possibly overlooked the diagnosis of Anti-NMDAR encephalopathy (42). Furthermore, the confounding impact of multidrug addiction on the manifestation of catatonia in this case deserves attention. Conventional body fluid toxicology tests for cannabinoids have limitations due to cannabis narrow toxicologic window, low effective dose, the lack of test sensitivity for new marijuana formulations with less THC and more CBD and synthetic cannabinoids (11, 47). Nevertheless, it is worth noting the significant temporal association with cannabis use or overuse, which warrants reporting. This case report, along with the review of existing literature, aims to emphasize the importance of comprehending and accounting for the substantial adverse effects associated with cannabis use and prescription. Future studies and discussions are imperative to gain a deeper understanding and quantify such burdening effects of cannabis.

### 3.4 Future directions

Some directions for future research can be to mechanistically study the substance-induced catatonia probably using computational approaches to inform quantitative and causal relation between cannabinoids and neural dysfunction including catatonic state. As the medical use of marijuana is a developing approach in neurological and psychiatric disorders, prompt animal or simulation studies are warranted to investigate how different formulations of cannabinoids differ in case of inducing catatonia or other major psychological sequelae. One way to root for the association between cannabis use disorder and catatonia is to look for a shared genetic substrate between the two. As prior study has attempted to look at genetic risk factors related to each condition (48, 49). This knowledge is crucial for predicting and identifying susceptible individuals or formulating safer prescriptions for medical cannabinoids, as well as for promptly recognizing motor or psychotic symptoms in patients with a history of cannabis use.

## 4 Conclusion

Finally, the intricate relationship between the use of cannabis and the manifestation of catatonia unveils a complex phenomenon that continues to captivate our understanding. While cannabis has demonstrated numerous therapeutic advantages, particularly in the treatment of movement disorders, the emergence of catatonia in specific individuals remains an enigmatic paradox. This perplexing enigma can be attributed to a multitude of factors, including individual susceptibility, the interplay of various cannabinoids, and the intricate modulation of neurotransmitter systems, encompassing the glutamatergic, GABAergic, and dopaminergic pathways. These multifaceted mechanisms, in conjunction with genetic and environmental variables, collectively shape the diverse range of responses to cannabis and underscore the imperative for further investigation.

## Data availability statement

The original contributions presented in the study are included in the article/Supplementary material, further inquiries can be directed to the corresponding authors.

## Ethics statement

The studies were conducted in accordance with the local legislation and institutional requirements. The participants provided their written informed consent to participate in this study. Written informed consent was obtained from the individual(s) for the publication of any potentially identifiable images or data included in this article.

## Author contributions

RM: Conceptualization, Supervision, Writing – original draft, Writing – review & editing. MH: Writing – original draft, Writing – review & editing. SM: Investigation, Writing – original draft, Writing – review & editing. RJ: Conceptualization, Visualization, Writing – review & editing. MM: Methodology, Software, Writing – original draft, Writing – review & editing. EC: Resources, Supervision, Validation, Visualization, Writing – original draft. JA: Project administration, Validation, Visualization, Writing – review & editing.

## References

[1] Tandon R Heckers S Bustillo J Barch DM Gaebel W Gur RE. Catatonia in DSM-5. Schizophr Res. (2013) 150:26–30. doi: 10.1016/j.schres.2013.04.034 23806583

[2] Taylor MA Fink M. Catatonia in psychiatric classification: a home of its own. Am J Psychiatr. (2003) 160:1233–41. doi: 10.1176/appi.ajp.160.7.1233, PMID: 12832234

[3] Edinoff AN Kaufman SE Hollier JW Virgen CG Karam CA Malone GW. Catatonia: clinical overview of the diagnosis, treatment, and clinical challenges. Neurol Int. (2021) 13:570–86. doi: 10.3390/neurolint13040057, PMID: 34842777 PMC8628989

[4] Palma-Alvarez RF Soriano-Dia A Ros-Cucurull E Daigre C Serrano-Perez P Ortega-Hernandez G. Catatonia related to cannabis and synthetic cannabinoids: a review. J Dual Diagn. (2021) 17:159–71. doi: 10.1080/15504263.2021.1904163, PMID: 33902405

[5] Yeoh SY Roberts E Scott F Nicholson TR David AS Rogers JP. Catatonic episodes related to substance use: a cross-sectional study using electronic healthcare records. J Dual Diagn. (2022) 18:52–8. doi: 10.1080/15504263.2021.2016342, PMID: 35001837

[6] Daniels J. Catatonia: clinical aspects and neurobiological correlates. J Neuropsychiatry Clin Neurosci. (2009) 21:371–80. doi: 10.1176/jnp.2009.21.4.371, PMID: 19996245

[7] Northoff G. What catatonia can tell us about “top-down modulation”: a neuropsychiatric hypothesis. Behav Brain Sci. (2002) 25:555–77. doi: 10.1017/S0140525X02000109, PMID: 12958742

[8] Zou S Kumar U. Cannabinoid receptors and the endocannabinoid system: signaling and function in the central nervous system. Int J Mol Sci. (2018) 19. doi: 10.3390/ijms19030833, PMID: 29533978 PMC5877694

[9] WHO. The use of the WHO–UMC system for standardised case causality assessment [Internet]. Geneva: World Health Organization (2020).

[10] Bajaj V Pathak P Mehrotra S Singh V Govil S Khanna A. Cannabis induced periodic catatonia: a case report. Int J Ment Heal Addict. (2011) 9:162–4. doi: 10.1007/s11469-009-9262-9

[11] Cohen J Morrison S Greenberg J Saidinejad M. Clinical presentation of intoxication due to synthetic cannabinoids. Pediatrics. (2012) 129:e1064–7. doi: 10.1542/peds.2011-1797 22430444

[12] Leibu E Garakani A McGonigle DP Liebman LS Loh D Bryson EO. Electroconvulsive therapy (ECT) for catatonia in a patient with schizophrenia and synthetic cannabinoid abuse: a case report. J ECT. (2013) 29:e61–2. doi: 10.1097/YCT.0b013e318290fa36, PMID: 23670023

[13] Haro G Ripoll C Ibáñez M Orengo T Liaño VM Meneu E. Could spice drugs induce psychosis with abnormal movements similar to catatonia? Psychiatry. (2014) 77:206–8. doi: 10.1521/psyc.2014.77.2.206, PMID: 24865202

[14] Smith DL Roberts C. Synthetic marijuana use and development of catatonia in a 17-year-old male. Minn Med. (2014) 97:38. 24941587

[15] Caudron M Rolland B Deheul S Geoffroy PA Thomas P Amad A. Catatonia and cannabis withdrawal: a case report. Subst Abus. (2016) 37:188–9. doi: 10.1080/08897077.2015.1052869, PMID: 26247767

[16] Håkansson A Johansson BA. Atypical course in severe catatonic schizophrenia in a cannabis-dependent male adolescent: a case report. J Med Case Rep. (2015) 9:1–5. doi: 10.1186/s13256-015-0678-5 26388066 PMC4576396

[17] Khan M Pace L Truong A Gordon M Moukaddam N. Catatonia secondary to synthetic cannabinoid use in two patients with no previous psychosis. Am J Addict. (2016) 25:25–7. doi: 10.1111/ajad.12318, PMID: 26781357

[18] Roberto AJ Lorenzo A Li KJ Young J Mohan A Pinnaka S. First-episode of synthetic cannabinoid-induced psychosis in a young adult, successfully managed with hospitalization and risperidone. Case reports. Psychiatry. (2016) 2016:1–4. doi: 10.1155/2016/7257489 PMC4939204 27429822

[19] Pierre JM Gandal M Son M. Cannabis-induced psychosis associated with high potency “wax dabs”. Schizophr Res. (2016) 172:211–2. doi: 10.1016/j.schres.2016.01.056, PMID: 26876313

[20] Keller CJ Chen EC Brodsky K Yoon JH. A case of butane hash oil (marijuana wax)–induced psychosis. Subst Abus. (2016) 37:384–6. doi: 10.1080/08897077.2016.1141153, PMID: 26820171

[21] Williams DR Miller BJ Tatugade A Avasthi R Buckley PF. Unresponsive and mute after he smoked ‘spice’. Curr Psychiatr Ther. (2016) 15:65.

[22] Bulbena-Cabre A DiGenova P Sigel P Dunn NR Swift RG. Synthetic cannabinoid intoxication presenting as malignant catatonia: a case report. Int J Ment Heal Addict. (2020) 18:582–6. doi: 10.1007/s11469-018-9954-0

[23] Manning T Bartow C McNaughton M Reynolds E Chen Z. Vaping cannabis oil: a case of catatonia associated with use of high-potency cannabis. Psychosomatics. (2020) 61:745–51. doi: 10.1016/j.psym.2020.06.012, PMID: 32771240

[24] Mekala H Malik Z Lone J Shah K Ishaq M. Cannabis-induced catatonia: a case series. Cureus. (2020) 12:6. doi: 10.7759/cureus.8603 PMC7362598 32676242

[25] Sheikh B Hirachan T Gandhi K Desai S Arif R Isakov O. Cannabis-induced malignant catatonia: a medical emergency and review of prior case series. Cureus. (2021) 13. doi: 10.7759/cureus.17490 PMC8437206 34548987

[26] Gouse BM Nieves-Archibald A Trutzer I Rezvani M Srinath M Chang A. Pediatric malignant catatonia associated with vaporized cannabis use: a case series. J Acad Consult Liaison Psychiatry. (2021) 62:445–8. doi: 10.1016/j.jaclp.2021.02.004, PMID: 34210403

[27] Palkar P Ray S. An atypical case of catatonia, abnormal gait and psychosis in an adolescent with chronic Cannabis use. J Drugs Addict Therapeut. (2021) 2:1–3. doi: 10.47363/JDAT/2021(2)114

[28] Gauthier T Prakash PB Drew Keopple N Vardis R. Cannabis-induced catatonia in a 15-year-old male: a case report. WMJ. (2023) 132 37141480

[29] Charlesworth JE Ghosn O Hussain N Mahmoud R Goncalves V Godbole M. A case report of an unusual presentation of a patient with recurrent idiopathic catatonia. Psychiatry Res Case Rep. (2023) 2:100111. doi: 10.1016/j.psycr.2023.100111

[30] Medda P Toni C Luchini F Giorgi Mariani M Mauri M Perugi G. Catatonia in 26 patients with bipolar disorder: clinical features and response to electroconvulsive therapy. Bipolar Disord. (2015) 17:892–901. doi: 10.1111/bdi.12348, PMID: 26643014

[31] Lin C-C Hung Y-Y Tsai M-C Huang T-L. Relapses and recurrences of catatonia: 30-case analysis and literature review. Compr Psychiatry. (2016) 66:157–65. doi: 10.1016/j.comppsych.2016.01.011, PMID: 26995249

[32] Lin C-C Huang T-L. Lorazepam–diazepam protocol for catatonia in schizophrenia: a 21-case analysis. Compr Psychiatry. (2013) 54:1210–4. doi: 10.1016/j.comppsych.2013.06.003, PMID: 23856388

[33] Fein S McGrath MG. Problems in diagnosing bipolar disorder in catatonic patients. J Clin Psychiatry. (1990) 51:203–5. PMID: 2335495

[34] Nath S Bhoi R Mishra B Padhy S. Does recurrent catatonia manifest in a similar fashion in all the episodes of mood disorder? A case series with literature review. Gen Psychiatry. (2021) 34. doi: 10.1136/gpsych-2021-100494 PMC8420649 34595400

[35] Bilbao A Spanagel R. Medical cannabinoids: a pharmacology-based systematic review and meta-analysis for all relevant medical indications. BMC Med. (2022) 20:259. doi: 10.1186/s12916-022-02459-1, PMID: 35982439 PMC9389720

[36] Oikonomou P Jost W. Randomized controlled trials on the use of cannabis-based medicines in movement disorders: a systematic review. J Neural Transm. (2022) 129:1247–56. doi: 10.1007/s00702-022-02529-x, PMID: 35859051

[37] Sánchez-Blázquez P Rodríguez-Muñoz M Garzón J. The cannabinoid receptor 1 associates with NMDA receptors to produce glutamatergic hypofunction: implications in psychosis and schizophrenia. Front Pharmacol. (2014) 4:169. doi: 10.3389/fphar.2013.00169 24427139 PMC3877778

[38] Sideli L Quigley H La Cascia C Murray RM. Cannabis use and the risk for psychosis and affective disorders. J Dual Diagn. (2020) 16:22–42. doi: 10.1080/15504263.2019.1674991 31647377

[39] Karniol I Carlini E. Pharmacological interaction between cannabidiol and Δ 9-tetrahydrocannabinol. Psychopharmacologia. (1973) 33:53–70. doi: 10.1007/BF00428793 4358666

[40] Rissardo JP Konduru SN Gadamidi VK Caprara ALF. Baclofen and catatonia: a case report. Pan Afr Med J. (2022) 43:43. doi: 10.11604/pamj.2022.43.198.38403 36942140 PMC10024567

[41] Ali HT Mohamed FR Al-Ghannami AK Caprara ALF Rissardo JP. Catatonia as the presentation of encephalopathy associated with autoimmune thyroiditis: a case report and literature review. J Psychiatr Pract. (2023) 29:499–504. doi: 10.1097/PRA.0000000000000751, PMID: 37948176

[42] Parenti A Jardri R Geoffroy PA. How anti-NMDAR encephalitis sheds light on the mechanisms underlying catatonia: the neural excitatory/inhibitory imbalance model. Psychosomatics. (2016) 57:336–8. doi: 10.1016/j.psym.2016.01.007, PMID: 27155123

[43] Rivas-Santisteban R Lillo A Lillo J Rebassa J-B Contestí JS Saura CA. N-Methyl-D-aspartate (NMDA) and cannabinoid CB 2 receptors form functional complexes in cells of the central nervous system: insights into the therapeutic potential of neuronal and microglial NMDA receptors. Alzheimers Res Ther. (2021) 13:1–15. doi: 10.1186/s13195-021-00920-6 34749800 PMC8576920

[44] Moshfeghinia R Oji B Hosseinzadeh M Pourfridoni M Ahmadi J. Early onset frontotemporal dementia following cannabis abuse: a case report. BMC Psychiatry. (2023) 23:484. doi: 10.1186/s12888-023-04956-w, PMID: 37391735 PMC10311823

[45] McPartland JM Duncan M Di Marzo V Pertwee RG. Are cannabidiol and Δ9-tetrahydrocannabivarin negative modulators of the endocannabinoid system? A systematic review. Br J Pharmacol. (2015) 172:737–53. doi: 10.1111/bph.12944, PMID: 25257544 PMC4301686

[46] Gadelho LS Marques JG. Catatonia associated with epileptic seizures: a systematic review of case reports. Epilepsy Res. (2022) 186:107016. doi: 10.1016/j.eplepsyres.2022.107016 36116265

[47] Johnson OE Miskelly GM Rindelaub JD. Testing for cannabis intoxication: current issues and latest advancements. Wiley Interdiscipl Rev. (2022) 4:e1450. doi: 10.1002/wfs2.1450

[48] Wilson JE Sealock J Straub P Raman R Kipp AM Dittus RS. Exploring genetic risk for catatonia in a genome wide association study and polygenic risk score analysis. Schizophr Res. (2023) 263:178–90. doi: 10.1016/j.schres.2023.07.015 37517919 PMC10822029

[49] Hillmer A Chawar C Sanger S D’Elia A Butt M Kapoor R. Genetic basis of cannabis use: a systematic review. BMC Med Genet. (2021) 14:1–21. doi: 10.1186/s12920-021-01035-5 PMC8359088 34384432

