# Supplementary material for: Recurrent cannabis-induced catatonia: a case report and comprehensive systematic literature review
Source: Front Psychiatry. 2024 Jan 18;15:1332310. doi: 10.3389/fpsyt.2024.1332310 (PMC10835799; doi:10.3389/fpsyt.2024.1332310)
Supplement: Supplementary file 1 [file Table_1.DOCX]

**Recurrent Cannabis-Induced Catatonia: A Case Report and Comprehensive Systematic Literature Review**

Reza Moshfeghinia^1,2,3†^, Mehrnaz Hosseinzadeh^4,5†^, Sara Mostafavi^1^, Roxana Jabbarinejad^6^, Mahdi Malekpour^1,2^, Elnaz Chohedri^2*^, Jamshid Ahmadi^3,7*^

^1^ Student Research Committee, Shiraz University of Medical Sciences, Shiraz, Iran

^2^ Research Center for Psychiatry and Behavioral Sciences, Shiraz University of Medical Sciences, Shiraz, Iran

^3^ Substance Abuse Research Center, Shiraz University of Medical Sciences, Shiraz, Iran

^4^ Fasa Neuroscience Circle (FNC), Student Research Committee, Fasa University of Medical Sciences, Fasa, Iran

^5^ National Brain Center, Iran University of Medical sciences,Tehran,Iran.

^6^ The Ken and Ruth Davee Department of Neurology, Northwestern University Feinberg School of Medicine, Chicago, Illinois, USA

^7^ Institute for Multicultural Counseling & Education Services (IMCES), Los Angeles, CA, US

*Corresponding authors:

1. Jamshid Ahmadi; [jamshid_ahmadi@yahoo.com](mailto:jamshid_ahmadi@yahoo.com); Institute for Multicultural Counseling & Education Services (IMCES), Los Angeles, CA, US
2. Elnaz Chohedri; [elnaz_chohedri@yahoo.com](mailto:elnaz_chohedri@yahoo.com); Research Center for Psychiatry and Behavioral Sciences, Shiraz University of Medical Sciences, Shiraz, Iran

† They are equal contributors to this work and designated as co-first authors
